# Lipid Saturation and Cholesterol Drive the Mechanical Response of Lipid Bilayer to Ionic Liquid: An Atomic Force Microscopy Study

**DOI:** 10.1021/acs.langmuir.5c06231

**Published:** 2026-03-14

**Authors:** Alyona Yedelkina, Brian J. Rodriguez, Alessandro Podestà, Antonio Benedetto

**Affiliations:** † School of Physics, 8797 University College Dublin, Dublin D04 N2E5, Ireland; ‡ Conway Institute of Biomolecular and Biomedical Research, University College Dublin, Dublin D04 N2E5, Ireland; § Department of Physics “Aldo Pontremoli”, University of Milano, Milan 20133, Italy; ∥ Department of Science, University of Roma Tre, Rome 00146, Italy

## Abstract

Ionic liquids (ILs) have recently emerged as promising amphiphilic electrolytes capable of modulating biomembrane properties, yet the mechanistic determinants underlying IL–lipid interactions remain insufficiently characterized. Here, we investigate the effect of the imidazolium IL [C_6_mim]­[Cl] on the structural and mechanical properties of phosphatidylcholine bilayers with controlled acyl-chain saturation (DPPC, POPC, DOPC) and cholesterol content. All measurements were performed at an IL concentration below the critical micellar concentration to avoid contributions from IL aggregation. Using atomic force microscopy, we show that even in the absence of topographical changes, the IL significantly affects membrane mechanics, as quantified by rupture force (RF) and Young’s modulus (YM), exhibiting a strong dependence on lipid saturation and cholesterol content. For DPPC-Chol, incubation in [C_6_mim]­[Cl] leads to a pronounced decrease in RF (−26 ± 9%) and YM (−30 ± 10%), while no effect is observed without cholesterol. For POPC, a pronounced increase in RF is observed (+30 ± 9%), which reverts in the presence of cholesterol (−13 ± 7%), which also softens the bilayer (−19 ± 9%). For DOPC, a pronounced increase in YM is observed (+30 ± 14%), which reverts with the addition of cholesterol (−27 ± 5%) that, surprisingly, leads to a pronounced increase in RF (+34 ± 9%). Control measurements with NaCl further confirm that the observed effects are not due to ionic strength alone, highlighting the critical role of the IL organic cation in modulating bilayer mechanics. Overall, these findings establish lipid saturation and cholesterol content as key variables governing IL–membrane interactions, and offer a framework for rationally tuning ILs to modulate membrane mechanics—relevant for nanobiotechnology, drug delivery, and membrane engineering.

## Graphical abstract

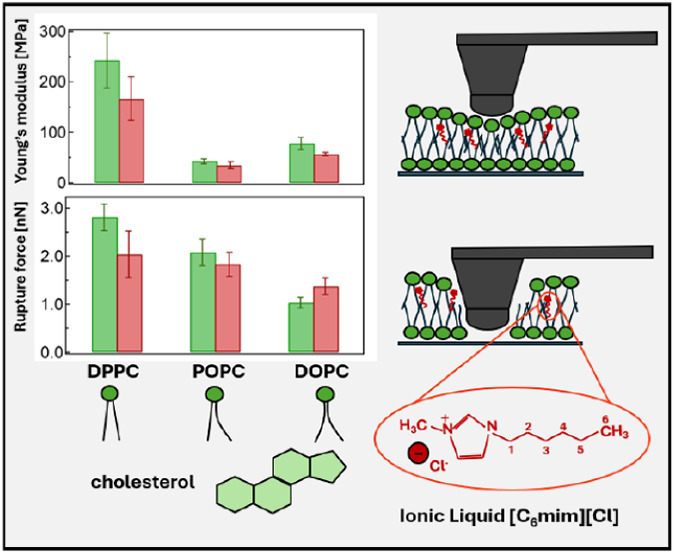

## Introduction

The cell membrane, a functional network of lipids and proteins, contains over a thousand different lipid species that vary in both headgroup and hydrocarbon chain composition. The latter strongly influences membrane fluidity, thereby defining membrane functionality and ultimately cell viability.^1,2^ Among membrane lipids, phospholipids—particularly phosphatidylcholines (PCs)—are the most abundant.^1,3^ These include saturated and unsaturated species, which play crucial roles in regulating membrane fluidity, phase behavior, and interactions with proteins and other biomolecules.^4^ Another major component of cell membranes is cholesterol. It plays a key structural and regulatory role by inserting into the hydrophobic lipid bilayer core, modulating the motion of hydrocarbon chains, and thereby controlling membrane fluidity and permeability.^1,4,5^ Cholesterol also contributes to the formation of lipid rafts—membrane nanodomains enriched in sphingolipids and cholesterol—which serve as platforms for protein organization, signaling, and metabolic processes.^6,7^

Ionic liquids (ILs) are organic electrolytes characterized by low melting points. Their highly tunable physicochemical properties and ability to interact with living systems have made them promising candidates for nanotechnological and biomedical applications.^8–13^ However, the mechanisms underlying their interactions with biological structures remain incompletely understood.^14–18^

Imidazolium-based ILs represent one of the most extensively studied classes of ILs. Their cations consist of an imidazole ring substituted with one or more hydrocarbon chains (Figure 1), while their anions are typically halides such as Cl^–^ or Br^–^. Owing to their amphiphilic nature—with a polar imidazolium headgroup and nonpolar alkyl tail—these ILs structurally resemble phospholipids and have been shown to partition preferentially into lipid membranes, embedding their hydrophobic tails within the bilayer core.^19,20^

**Figure 1:**
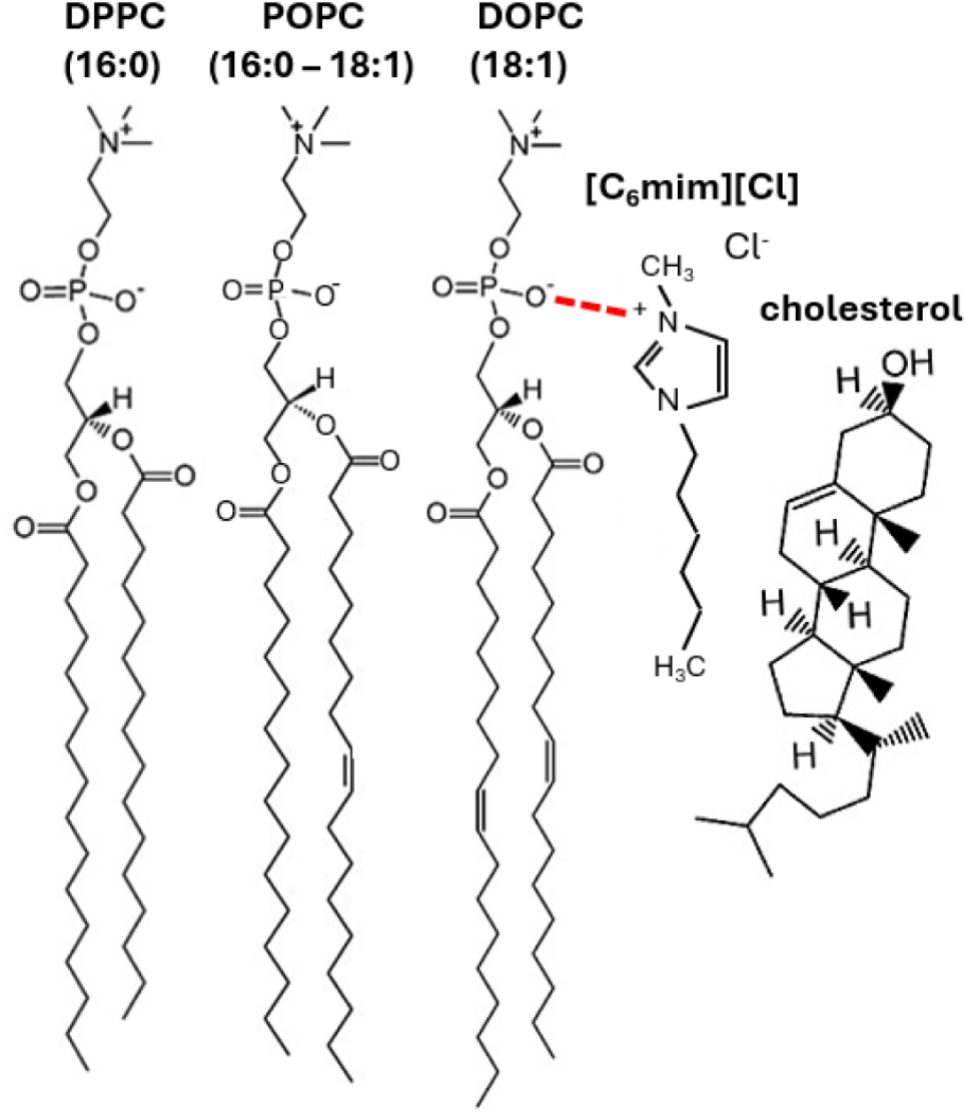
Chemical structures of phosphatidylcholines DPPC, POPC, and DOPC, cholesterol, and [C_6_mim]­[Cl] IL used in this study. Based on MD studies,^31^ the positive charge of the IL imidazolium cation is paired with one electronegative oxygen atom of the lipid phosphate group (sketched in dashed red for clarity for DOPC-IL only), and the IL tail is aligned with the lipid hydrocarbon chain. The relative height is preserved, which also highlights the length mismatch between lipid and IL tails.

This structural similarity has motivated extensive research into the interactions between ILs and biological membranes.^21,22^ For example, Kumari et al. demonstrated that [C_4_mim]­[Cl] and [C_10_mim]­[Cl] ILs can reduce cell membrane elasticity even at subtoxic concentrations, affecting the mobility of cancerous MDA-MB-231 cells. This effect was shown to depend on both the alkyl chain length and the concentration of the IL.^23^ Further studies have explored the impact of ILs on the lipid phase transition temperature,^24,25^ membrane stability and elasticity,^26,27^ its structural and dynamic properties,^28–32^ as well as cell morphology and rigidity.^33^ A comprehensive overview of these findings can be found in two reviews by A. Benedetto,^21,22^ which summarize the major advances in the study of IL–cell membrane interactions since the earliest reports in this field.

The interest of studying IL–membrane interactions is motivated by the fact that the cell membrane is the first point of contact for any external agents, serves as a barrier that maintains cellular homeostasis and takes a key part in a variety of critical cellular functions. Its composition and structural features vary between cell types, providing opportunities for targeted interactions. Understanding these mechanisms can therefore provide insight into how ILs affect different cells and organisms, and may inform strategies for targeted therapies, including selective treatment of cancerous or bacterial cells.

While much of the existing research has focused on how ILs properties—such as alkyl chain length, cation and anion identity, and concentration—govern IL–membrane interactions,^29,34^ comparatively little attention has been given to the role of membrane lipid properties themselves.^35,36^ Understanding how intrinsic lipid characteristics modulate IL interactions is essential for predicting IL effects in more complex membrane environments and, ultimately, for extending these insights to the level of cellular membranes.

In this work, we address this gap by investigating how different lipid saturation modulates the interaction between the model imidazolium IL [C_6_mim]­[Cl] and model phospholipid membranes. We focus on three representative PCs with distinct degrees of unsaturation: DPPC (fully saturated), POPC (asymmetrically unsaturated), and DOPC (symmetrically unsaturated), all of which are commonly found in mammalian cell membranes. The presence or absence of unsaturated bonds is one of the factors defining membrane fluidity.^4^ ILs, known to interact with lipid bilayers by inserting their hydrocarbon chains into the hydrophobic membrane core, are able to alter interactions between lipid chains and hence membrane structural and mechanical properties.^19^ In this context, the IL [C_6_mim]­[Cl] was selected based on previous evidence indicating that its alkyl chain length enables penetration beneath the interfacial zone of lipid bilayers, allowing interactions with their hydrophobic cores.^37^ Figure 1 reports the chemical structures of the three PC lipids and the IL, highlighting the representative IL–lipid configuration and the length mismatch between the lipid and IL tails, which plays a key role in the IL–lipid bilayer interaction.

In the latter part of the study, we further explore the role of cholesterol by examining its effect on each of these lipid systems under IL exposure. We used 20 mol % cholesterol in the model membranes to maintain a biologically relevant cholesterol concentration.^38^

Each of the model systems was treated with 10% CMC (critical micelle concentration) [C_6_mim]­[Cl] IL to avoid contributions from IL aggregation^39^ and prevent membrane destruction,^40^ as the aim of the study was to examine the effect of the IL on mechanical properties while preserving the structural integrity of the membranes.

By analyzing the topographical and mechanical properties of these model membranes with atomic force microscopy (AFM), we reveal how lipid saturation and cholesterol content influence [C_6_mim]­[Cl]-membrane interactions, providing new insight into the biophysical effects of imidazolium ILs.

## Materials and Methods

### Materials

Calcium chloride (CaCl_2_), sodium chloride (NaCl), and chloroform (CHCl_3_, >99% purity) were purchased from Sigma-Aldrich. Imidazolium ionic liquid, 1-hexyl-3-methylimidazolium chloride ([C_6_mim]­[Cl], >98% purity, IOLITEC (Ionic Liquid Technologies)) was used without further purification. Chloroform solutions of dipalmitoylphosphatidylcholine (16:0 DPPC), 1-palmitoyl-2-oleoylphosphatidylcholine (16:0–18:1 POPC), dioleoylphosphatidylcholine (18:1 DOPC), and cholesterol (C_27_H_46_O) powder with purity >98% were purchased from Avanti Polar Lipids. An extruder kit, 100 nm polycarbonate membranes, and filter supports were purchased from Avanti Polar Lipids. Muscovite mica disks (12 mm in diameter, Merck), Araldite instant liquid adhesive (Radionics LTD), Milli-Q water (electrical resistivity of 18.2 MΩ·cm), glass coverslips (Sigma-Aldrich, 20 mm × 20 mm), Petri dishes (Falcon, 35 mm diameter), and DNP-10 AFM probes (Bruker) with 0.12–0.35 N/m spring constant range (lever A and B) and a nominal tip radius of 20 nm were used.

### Supported Lipid Bilayer Preparation

For single-component lipid bilayers, 30 μL of the corresponding lipid chloroform solution was used. For cholesterol-containing bilayers, the corresponding chloroform solutions of the phospholipids were mixed with cholesterol in a molar ratio of 4:1. Cholesterol powder was first dissolved in chloroform to a final concentration of 33.33 mg/mL and stored at −20 °C until use. To remove chloroform, the solution was dried under a gentle stream of nitrogen and left under vacuum overnight. The dried lipid film was rehydrated with 1 mL of 10 mM CaCl_2_ buffer, heated to 50 °C, and vortexed. Twenty-one extrusion cycles at 50 °C (until a clear solution was obtained) were performed to obtain 100 nm lipid vesicles. The supported lipid bilayer was prepared on a mica disc attached to a glass coverslip. 150 μL of lipid vesicle solution with a final concentration of 0.1 mg/mL was pipetted onto freshly cleaved mica and incubated at 50–60 °C for 45 min, after which the sample was left to cool slowly to room temperature. The sample was then washed thoroughly with 10 mM CaCl_2_ buffer at room temperature and placed in a Petri dish filled with 10 mM CaCl_2_ buffer. The buffer was chosen based on an optimization procedure to ensure the preparation of a stable and reproducible supported lipid bilayer suitable for AFM measurements.

### Atomic Force Microscopy

AFM measurements on the lipid bilayer were conducted using a DriveAFM (Nanosurf, Switzerland) mounted on an Axio Observer 7 inverted optical epi-fluorescence microscope (Zeiss, Germany). The bilayer sample, placed in a Petri dish, was secured in a Nanosurf-designed Petri dish holder, and the entire setup was enclosed in an acoustic isolation chamber. The Petri dish holder featured temperature control. Measurements were conducted at 25 °C, which was maintained by setting the Petri dish holder to 25 °C. Before each experiment, the AFM system and the measured sample were allowed to equilibrate to the experimental temperature for at least 30 min.

Imaging and force spectroscopy measurements were performed using DNP triangular probes (Bruker, Germany) with a nominal resonance frequency ranging from 18 kHz to 23 kHz and spring constant ranging from 0.35 N/m to 0.12 N/m, respectively, and with a nominal apex radius of 20 nm. For imaging, the AFM was operated in WaveMode (WaveMode),^41^ an off-resonance tapping mode in which the cantilever was photothermally actuated via a laser excitation of 1–3 kHz frequency, resulting in a typical free-air oscillation amplitude of 10–15 nm; a maximum force set point of 300 pN was used. The typical scan size, scan rate, and number/line were set at 10 μm × 10 μm, 1.5 Hz, and 256 points, respectively. Force spectroscopy was performed in contact mode as a force map (force volume) on a 15 × 15 grid over a 10 μm × 10 μm area at a loading rate of 60 nN/s. A single force curve was acquired at each point of the grid.

Typical raw force spectroscopy data are obtained as the photodetector deflection signal, measured in volts (V), versus the piezoelectric Z-displacement, recorded in nanometers (nm). To convert the data into interaction force (nN) versus tip–substrate distance (nm) dependence, we add a cantilever deflection in nm to the piezoelectric Z-displacement, after multiplying the photodetector deflection signal by the deflection sensitivity (nm/V) to obtain tip–substrate distance; the force axis is obtained by multiplying the deflection by the probe spring constant (N/m).^42^ The spring constant is calculated by acquiring the thermal spectrum of the cantilever at the beginning of each AFM experiment and applying the Sader method.^43^ The deflection sensitivity is obtained using data analysis code in MATLAB from a force curve as the inverse of the slope of the contact part after the probe breaks the bilayer and reaches the underlying hard substrate (CD in Figure 2). A detailed description of the general calibration procedure can be found elsewhere.^42,44,45^ The resulting rescaled force–distance curve is shown in Figure 2 and represents the approaching-retracting cycle an AFM tip performs at each point of the force map.

**Figure 2:**
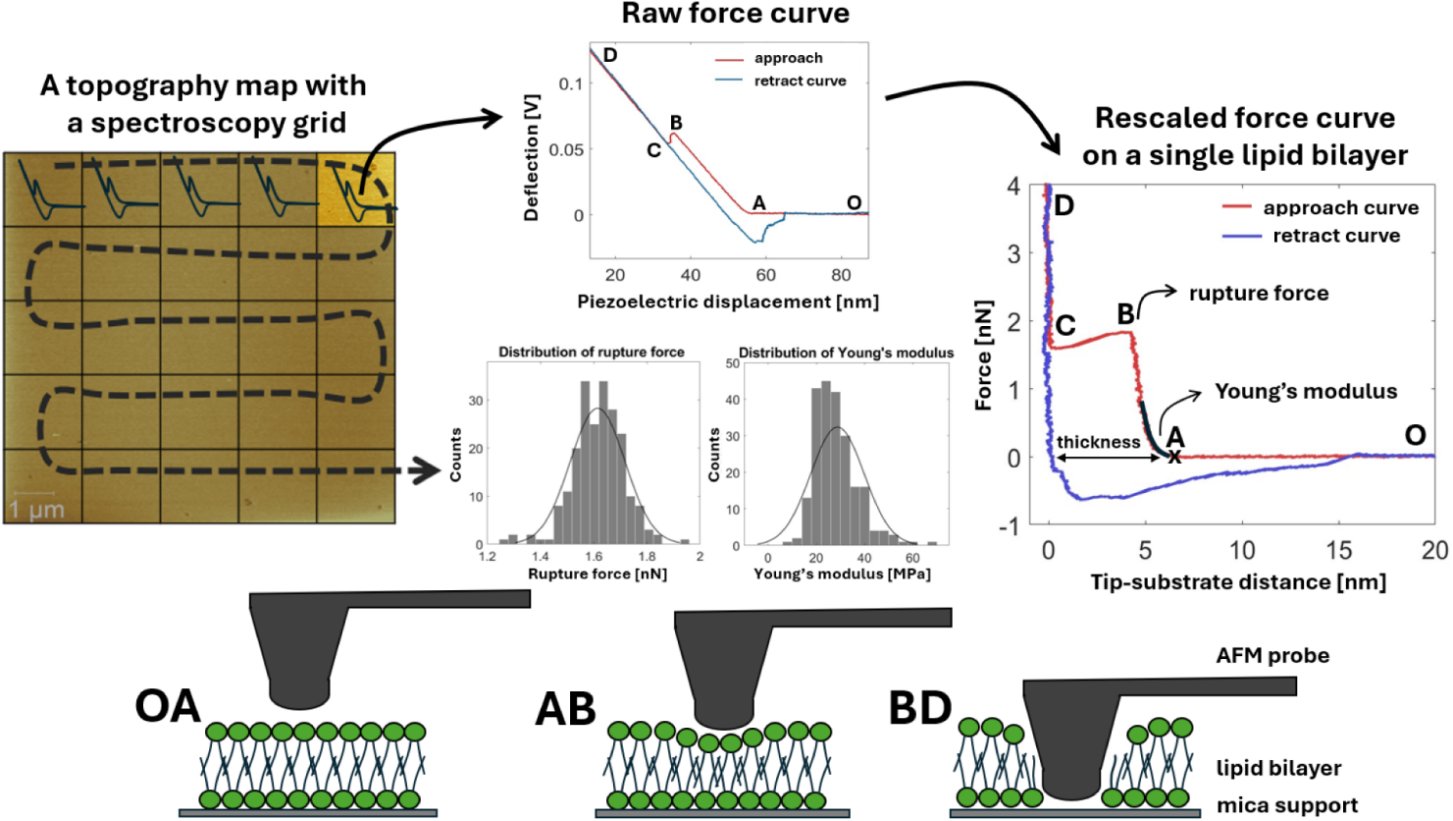
A representative force map and a force–distance curve obtained on a single lipid bilayer (pristine POPC sample) from each point of the map. The diagram illustrates the key stages of the indentation process: OA—the AFM probe is far from the membrane surface, no interaction force is detected; A—the initial point of contact between the probe tip and the lipid bilayer; AB—the bilayer deformation region; B—the rupture point of the bilayer; C—the point of tip contact with the underlying substrate; CD—the tip–substrate contact region. The force at point B is referred to as a rupture force and represents the bilayer’s mechanical strength. Fitting the AB portion of the curve using the Hertz model provides the bilayer’s Young’s modulus, a measure of its bending elasticity.

The force spectroscopy tip cycle starts far enough from the bilayer’s surface, where no interaction forces are detected (OA in Figure 2). By approaching, the tip experiences the attractive force that finally brings it into contact with the sample (A in Figure 2). Further tip movement produces deformation in the bilayer (AB) until it reaches the point of failure—the bilayer’s rupture (B). When going through the bilayer, the tip encounters less resistance, which results in a force decrease (BC) until the tip reaches the supporting substrate (C). The force at point B is referred to as a rupture force (RF) and is the measure of the bilayers’s strength and mechanical stability. The elastic portion of the AB curve in Figure 2 is fitted with the Hertz model to extract Young’s modulus (YM) of the lipid bilayer. The distance between tip–sample contact (A) and tip–substrate contact (C) is equal to the bilayer’s thickness.

Given that the tip apex has a radius of approximately 20 nm, its presses on an area of approximately 1260 nm^2^. Considering that 50–70 Å^2^ is the average area per lipid,^46,47^ we estimate that the tip stresses an area of about 2,500 lipid molecules. This suggests that our measurements reflect the properties of the membrane as a whole, rather than just a small assembly of a few lipid molecules.

### Experimental Protocol, Data Analysis, and Statistics

To assess the effect of the imidazolium IL on the model bilayers, we examined about 22 samples (3 each of DPPC and DOPC-cholesterol, 4 each of POPC, DOPC, DPPC-cholesterol, and POPC-cholesterol), each of which was measured in a pristine state in a 10 mM CaCl_2_ buffer first and then in the IL-containing solution, which was prepared by dissolving the IL in 10 mM CaCl_2_ to maintain the same concentration of CaCl_2_ throughout the experiment. After collecting a topography image and force spectroscopy map on a pristine sample, the CaCl_2_ buffer was exchanged with IL solution, and the sample’s topography and force spectroscopy data were collected every 15 min during the first hour of incubation in the IL, resulting in 5 sets of topography images and force maps for each of the 22 samples. Additionally, for each membrane composition, we performed control measurements with NaCl solution of the same ionic strength as [C_6_mim]­[Cl] (0.09 M, dissolved in 10 mM CaCl_2_) using the same experimental protocol. The aim was to isolate and evaluate the ionic contribution to the overall IL effect on lipid membranes.

Bilayer topography images were postprocessed using Gwyddion software,^48^ while force spectroscopy data were analyzed with a custom MATLAB script.^27^ The script enables extracting RFs by analyzing the histogram of distances in the force curve. The distance axis of the force curve is then transformed to the indentation axis by shifting the contact point to zero. YM is extracted by fitting the force–indentation curve with the Hertz model for a spherical indenter:

1 $$F=\frac{4}{3}\frac{E}{1-\nu^{2}}\sqrt{R}h^{3/2}$$

where *E* is Young’s modulus, *F* is the loading force, *h* is the indentation depth, *R* is the radius of the AFM probe, and ν is Poisson’s ratio, which is considered to be equal to 0.5 for incompressible biological samples.

The Hertz model assumes a sample that is homogeneous, isotropic, extends infinitely in all planar directions and depth, and exhibits a linear elastic response—conditions that are rarely met by biological samples.^49^ However, it has been shown that when the probe radius is at least ten times smaller than the sample’s lateral dimensions, and the indentation depth is much smaller than the probe radius, the sample can be approximated as an elastic half-space, making it suitable for applying Hertzian mechanics.^50^ While the presence of a rigid underlying substrate influences the absolute magnitude of the apparent YM (the bottom effect),^51^ our analysis focuses primarily on the relative differences between the pristine and IL-doped states, which are not influenced by this substrate-related effect. Figure S10 reports YM values corrected for the bottom effect, showing that the correction reduces the absolute modulus values by approximately 60%. The figure also compares the relative variations between the neat and IL-doped states for both the uncorrected and corrected data. Importantly, once the analysis is based on relative differences, the influence of the bottom-effect correction becomes negligible. Expressing the results in relative terms additionally minimizes the impact of experimental factors that can affect the absolute values, such as tip radius, humidity, and minor variations in sample preparation.

For each force map, distributions of RF and YM values were extracted and fitted with Gaussian functions to determine their mean and standard deviation. The effect of the IL at different incubation times was evaluated by normalizing the mean and standard deviation of the RF and YM obtained from the force map measurements on IL-treated membranes to the corresponding mean values of their pristine state. For each membrane sample, we acquired five force maps—one pristine (time zero) and four IL-treated (incubation time = 15, 30, 45, and 60 min)—from which the time-dependence of RF and YM was extracted. Each experimental condition was repeated 3–4 times to ensure reproducibility, yielding 3–4 individual time-dependent RF and YM curves. To obtain a single average trend, these individual time-dependent curves were averaged point by point, and the uncertainty at each time point was calculated as the standard error of the corresponding normalized RF or YM across all replicates. Because both RF and YM reached a plateau after approximately 30 min of incubation, the overall IL effect was determined by averaging the data collected at 30, 45, and 60 min.

## Results and Discussion

### Influence of Lipid Saturation on IL–Membrane Interactions

To investigate how lipid chain saturation influences IL–membrane interactions, we prepared three one-component lipid systems composed of DPPC, POPC, or DOPC, representing fully saturated, asymmetrically unsaturated, and symmetrically unsaturated molecules, respectively. Each system was incubated with 10% CMC [C_6_mim]­[Cl] to prevent membrane disruption, as our aim was to examine the mechanical effects of the IL on structurally intact lipid bilayers. The total incubation time was 60 min, with measurements taken every 15 min to evaluate the time dependence of IL-induced topographical and mechanical changes.

The first lipid studied was DPPC (*T*_m_ = 41 °C), which forms a model membrane in the gel phase (so) at the experimental temperature of 25 °C. This phase is characterized by a tightly packed, ordered bilayer structure with relatively slow lateral lipid mobility. The addition of [C_6_mim]­[Cl] resulted in only minor changes in the bilayer’s mechanical strength (RF) and elasticity (YM), both reaching a plateau after approximately 30 min. This behavior was reproducible across three independent replicates, with the average change in RF and YM between 30 and 60 min being +6 ± 5% and −7 ± 8%, respectively (Figure 3A,B).

**Figure 3:**
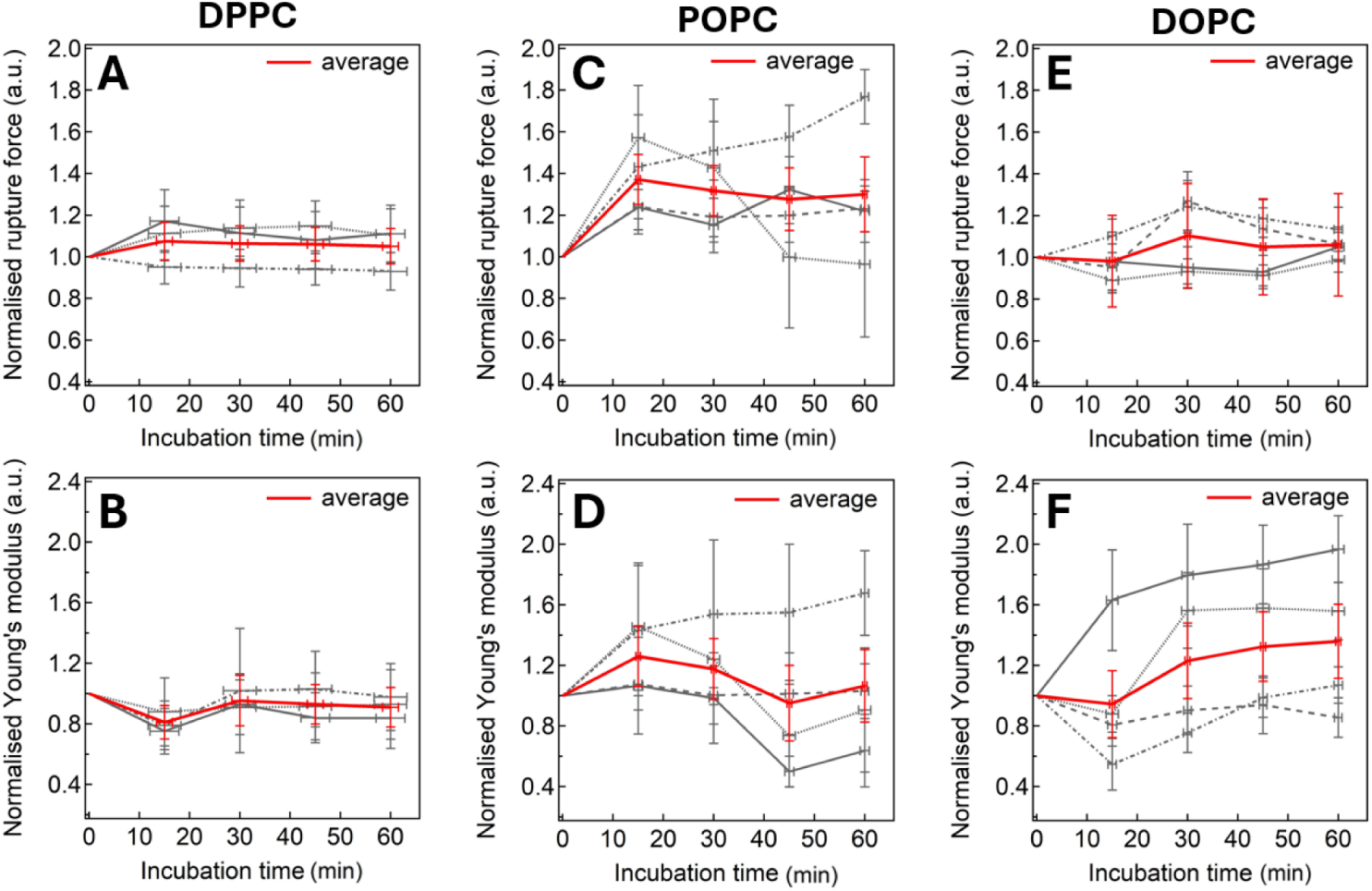
Time dependence of [C_6_mim]­[Cl]-induced changes in 100% PC membranes. Normalized RF (upper row) and YM (lower row) are shown. Panels A and B: DPPC; C and D: POPC; E and F: DOPC. The average response across replicates is plotted in red.

No significant topographical alterations were observed during the incubation period (Figure S1A). However, in some cases, small holes or protrusions appeared, following the pattern of the force spectroscopy grid and occurring only in the presence of [C_6_mim]­[Cl] (Figure S2A). Despite these localized features, all three replicate samples exhibited consistent RF and YM.

With melting temperatures below 0 °C, POPC (*T*_m_ = −2 °C) and DOPC (*T*_m_ = −17 °C) form membranes in the liquid-disordered (Ld) phase, characterized by highly fluid lipid molecules with rapid lateral diffusion.^52^ The addition of the IL to POPC or DOPC membranes did not induce any detectable disruption of membrane integrity (Figures S1B,C, S2B,C). Although no visible topographical alterations were observed, [C_6_mim]­[Cl] nevertheless modified the mechanical response of both membranes.

For POPC—structurally positioned between DPPC and DOPC—the IL induced a noticeable increase in mechanical stability, increasing its RF (Figure 3C). At the same time, the average YM of the POPC bilayer showed no major change. However, the IL effect on POPC was less reproducible than in the DPPC system, exhibiting varying behavior among replicates, from stiffening to softening (Figure 3D). On average, the changes in RF and YM were +30 ± 9% and +6 ± 13%, respectively.

Finally, the DOPC membrane exhibited the largest response to [C_6_mim]­[Cl], showing a significant stiffening of +30 ± 14% (YM), accompanied by a smaller rise of +7 ± 5% in the bilayer’s RF.

### Modulation of IL–Membrane Interactions by Cholesterol

Before addressing the effect of the IL on cholesterol-containing systems, we first examined the mechanical influence of cholesterol itself by measuring and comparing the absolute values of RF and YM of pristine bilayers with and without cholesterol at 25 °C (Figure 4).

**Figure 4:**
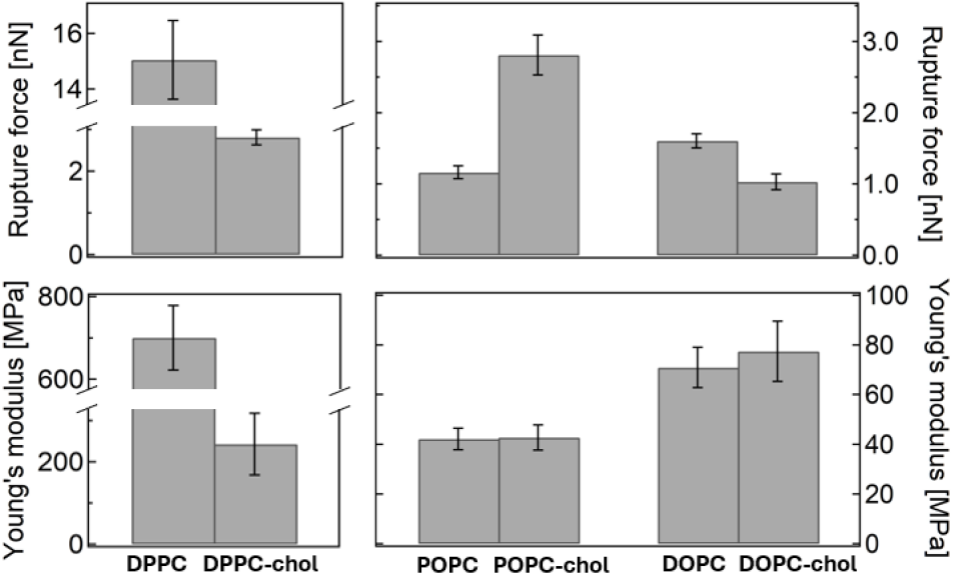
Comparison of absolute RF and YM in membranes with and without cholesterol.

The addition of cholesterol to DPPC resulted in a pronounced softening and structural weakening of the bilayer compared to its one-component counterpart. According to its phase diagram,^53^ the system undergoes a so-L_o_ phase transition under these experimental conditions, which explains the observed decrease in both RF and YM. Subsequent incubation with 10% CMC [C_6_mim]­[Cl] led to further weakening and softening of the DPPC–cholesterol system (Figure 5A,B). Interestingly, this membrane composition displayed distinct “populations” based on their mechanical response to the IL. In this context, ″populations″ are defined as groups of samples whose differences in normalized RF and YM are greater than the associated error bars. While the observed variation in mechanical response may originate from multiple factors (differences in the preparation protocol, changes in tip radius, sample spatial heterogeneity, or phase coexistence) and additional studies are required to fully understand these effects, our data acquisition protocol provides an average value, thereby allowing us to identify the overall effect of the IL. The average decreases in RF and YM were −26 ± 9% and −30 ± 10%, respectively. The membrane topography remained unchanged throughout the entire incubation period (Figure 6A, Figure S3A).

**Figure 5:**
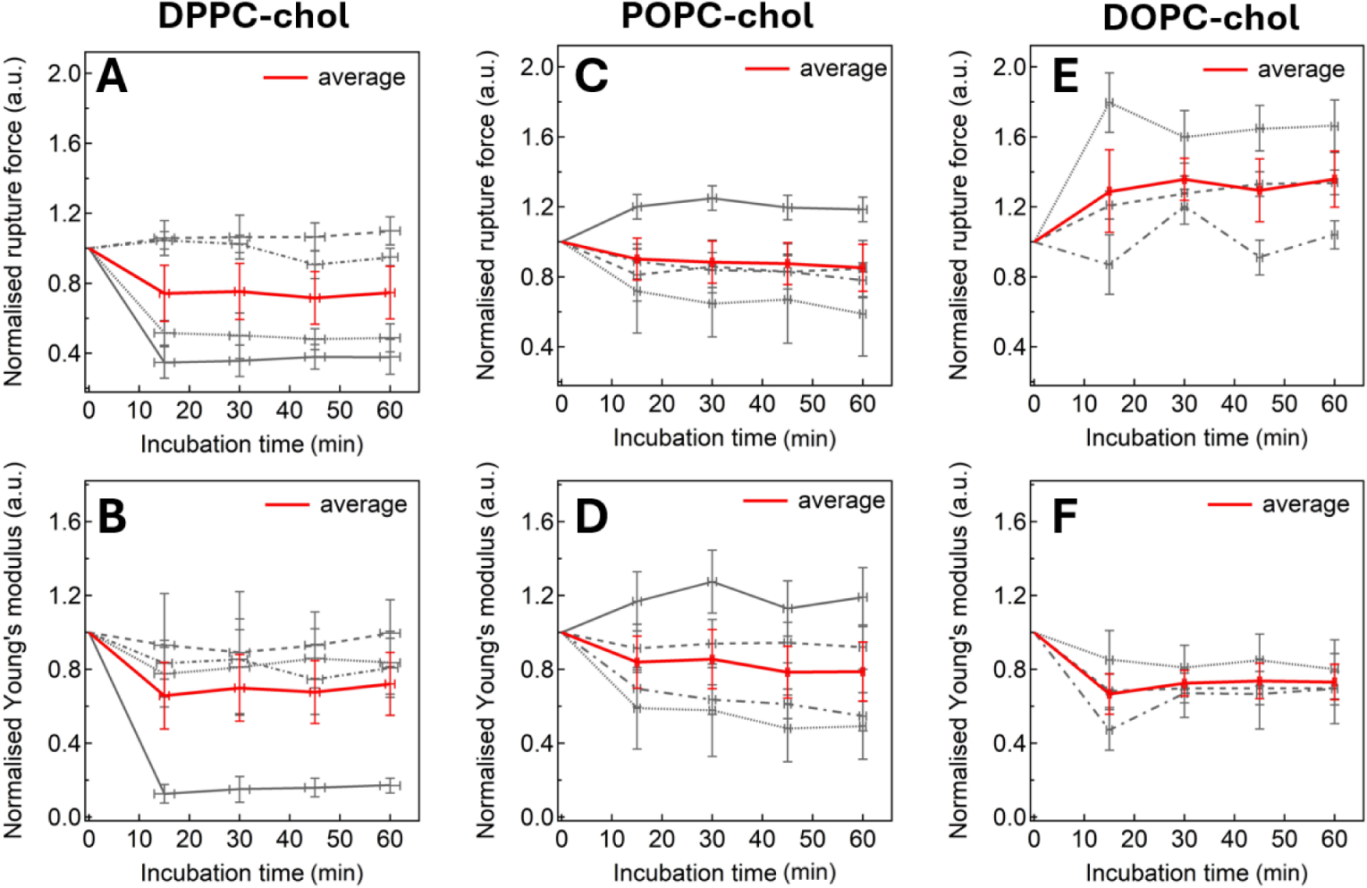
[C_6_mim]­[Cl]-induced changes in PC–cholesterol membranes over time. Normalized RF (top) and YM (bottom) are shown. Panels A–B: DPPC–cholesterol; C–D: POPC–cholesterol; E–F: DOPC–cholesterol. The mean across replicates is plotted in red.

**Figure 6:**
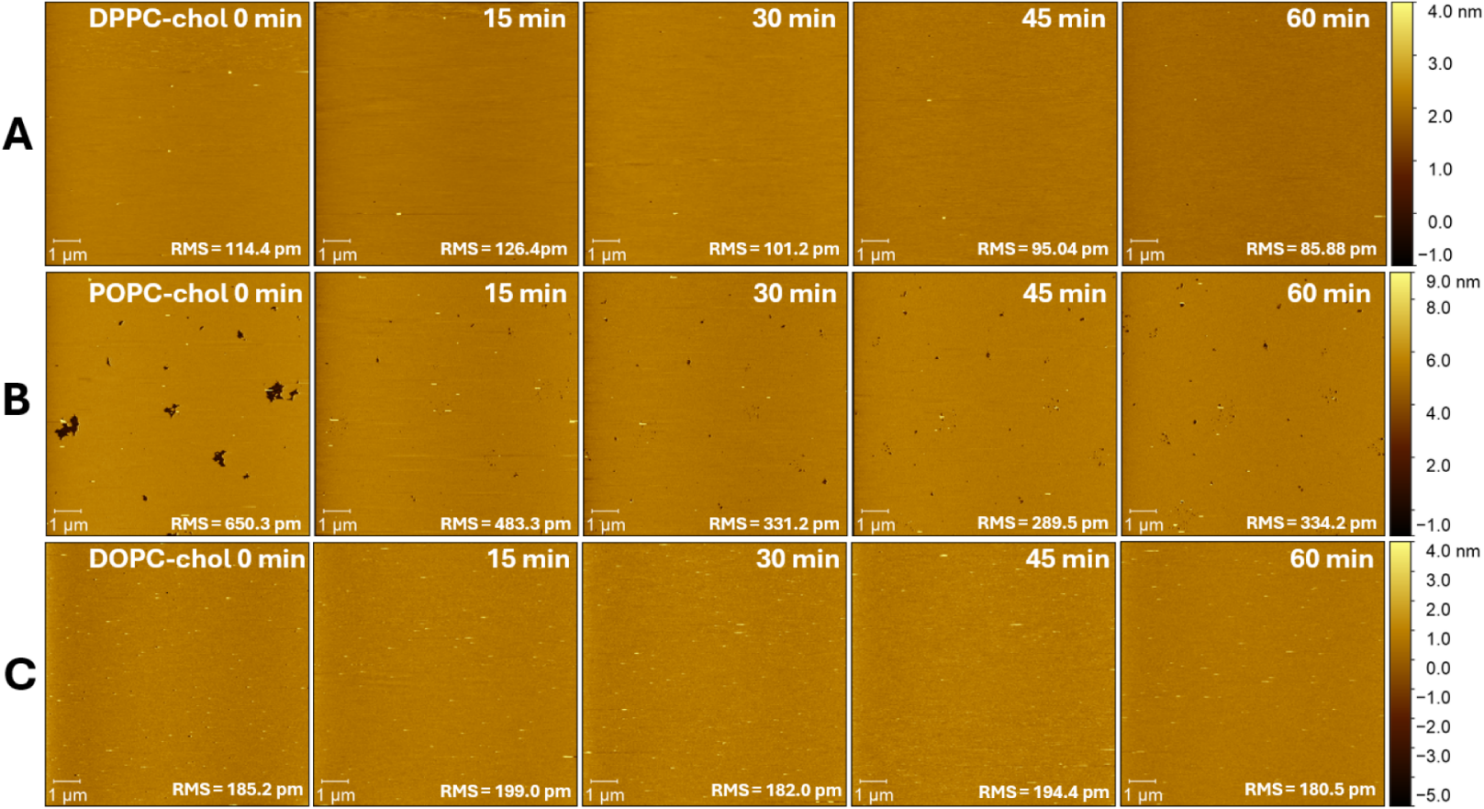
Effect of [C_6_mim]­[Cl] on the topography of PC–cholesterol membranes over 0–60 min. Panels A–C correspond to DPPC–cholesterol (A), POPC–cholesterol (B), and DOPC–cholesterol (C), respectively. The RMS roughness is provided to quantify the height variations across the surface.

In the pristine POPC and DOPC systems, cholesterol significantly affected mechanical stability (RF), whereas the elasticity (YM) remained unchanged within experimental uncertainty (Figure 4). Based on the corresponding phase diagrams,^54,55^ the incorporation of 20 mol % cholesterol induces the coexistence of so and L_o_ phases in the POPC–cholesterol system, increasing its mechanical stability (RF), whereas the DOPC–cholesterol membrane remains in the Ld state. The RF of DOPC–cholesterol, however, was lower than that of pure DOPC under the same experimental conditions, following the same trend observed in DPPC upon the addition of cholesterol. RF depends on multiple factors and the mechanisms behind its reduction may differ between DPPC and DOPC. In DPPC, for example, RF decreases due to disruption of the tightly packed gel phase due to cholesterol addition. In DOPC, on the other hand, it may result from the rigid cholesterol ring accommodating different tilt angles, which may relax lipid–lipid cohesion and surface molecular density.

In the presence of [C_6_mim]­[Cl], the RF of the POPC–cholesterol system decreased by −13 ± 7%, while that of DOPC–cholesterol increased by +34 ± 9% (Figure 5C,E). In both cases, however, the membranes became softer after IL exposure, with corresponding decreases of −19 ± 9% and −27 ± 5% in YM (Figure 5D,F). Consistent with the DPPC–cholesterol system, the POPC–cholesterol bilayer also exhibited distinct populations in its mechanical response. In contrast, no significant topographical changes were observed across all measured replicates (Figure 6B–C, Figure S3B–C). The POPC–cholesterol membrane presented same small preexisting defects that, following IL treatment, disappeared, in agreement with the softening effect of the IL (Figure 6B, Figure S3B).

### Electrostatic Control: NaCl-Induced Membrane Response

ILs interact with lipid membranes through both electrostatic interactions and their organic components. To rule out the possibility that the mechanical changes observed with [C_6_mim]­[Cl] arose solely from electrostatic effects, we performed a series of control experiments using NaCl solutions of equivalent ionic strength. All six lipid compositions were incubated with 0.09 M NaCl for 60 min, and topographical (Figures S4, S5) and mechanical measurements (Figures S6, S7) were collected every 15 min following NaCl addition. Figure 7 compares the average time-dependent effects of the IL and NaCl.

**Figure 7:**
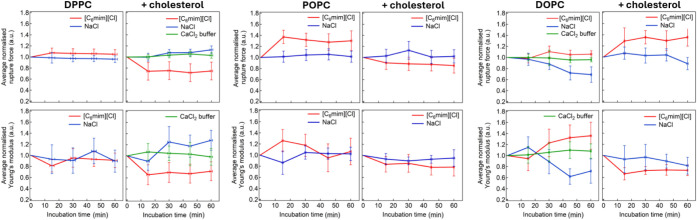
Comparison of NaCl- and [C_6_mim]­[Cl]-induced changes in membrane mechanical properties across all lipid compositions.

In most cases, the presence of NaCl caused no significant alterations in the bilayer properties, except for +24 ± 13% stiffening (YM) observed in the DPPC–cholesterol membrane, which is the opposite effect to the one observed with the IL. Similarly, for the DOPC membrane, NaCl had an effect opposite to that of the IL: it reduced both mechanical stability (RF) and elasticity (YM) (Figure 7).

The time dependence of RF and YM for all replicates of the NaCl-incubated samples, along with their averages, is presented in the (Figures S6 and S7). Topographical imaging revealed no major morphological changes (Figures S4, S5).

Overall, the ionic effect was negligible in most cases, suggesting that the IL effect arises from the complex interplay of both its electrostatic and organic nature. In the few systems where deviations were observed (DPPC–cholesterol and DOPC), additional control experiments were performed to verify bilayer stability. Specifically, the CaCl_2_ buffer was exchanged with an identical solution, and the topographical and mechanical properties were monitored for 3–4 replicates over the course of 1 h following the exchange. The results confirmed the absence of any detectable changes in bilayer strength (RF), elasticity (YM), or topography (Figure 7, Figure S8).

Average variations in RF and YM across all bilayers after incubation with IL and NaCl are summarized in Tables S1 and S2. Although the quantitative variations in RF and YM are reported above, representative average force curves are provided in Figure S9 to illustrate the overall mechanical response and facilitate visual comparison between the pristine and treated states. Figure S10 illustrates the impact of the bottom-effect correction.^51^ The results confirm that omission of this correction introduces no significant differences in the normalized data reported herein.

### Discussion

Two key mechanical parameters—RF and YM—were monitored, as both provide complementary information on the bilayer’s internal structure and intermolecular interactions. RF, defined as the critical force a bilayer can withstand before failure, has been correlated with lipid tail orientational order, internal stress distribution, molecular surface density, and the energy associated with the pore formation.^56–58^ YM, reflecting membrane stiffness, depends on the area per lipid and thus reports on the conformational freedom available mainly to lipid tails.^59^ Consequently, any perturbation that disrupts the membrane’s internal order is expected to increase the conformational space accessible to lipid tails, enhance lipid mobility, and reduce elasticity.^59^ Although these two parameters provide complementary information, they reflect independent processes and do not necessarily correlate. In other words, a stiffer membrane is not always easier to rupture—an effect that is indeed observed in our study for the DOPC-cholesterol system.

Figure 8 summarizes the main results of this study, comparing the effect of the IL (and NaCl) on the bilayers’ RF and YM.

**Figure 8:**
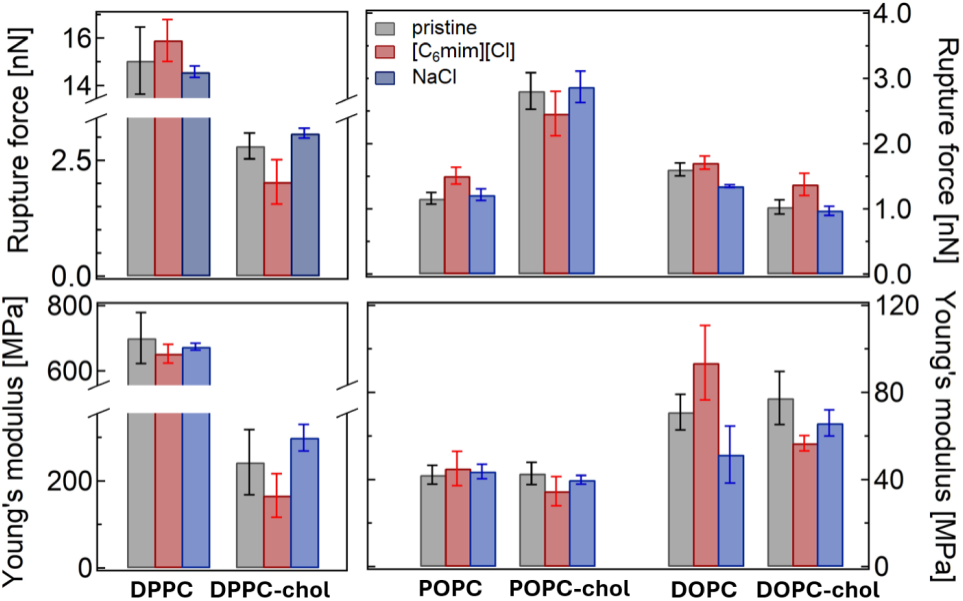
Average RF and YM values measured for each studied membrane composition in its pristine state and after 30–60 min incubation with 0.09 M [C_6_mim]­[Cl] or 0.09 M NaCl at 25 °C.

The effect of imidazolium ILs on lipid membranes depends on multiple factors, including IL partitioning, alkyl chain length, concentration, and the lipid composition of the membrane, which determines its physical properties (e.g., area per lipid and thermodynamic state). Concerning the IL partitioning between the lipid and the aqueous phases, for example, it is expected that the greater the IL amount in the lipid phase, the greater its effect on the bilayer’s properties. Previous studies have shown that IL partitioning into the lipid gel phase is significantly hindered compared with the liquid phase, due to the high energy barrier ILs must overcome to insert into a well-ordered bilayer.^60^ This phenomenon has been demonstrated primarily for saturated lipids, and the behavior may differ for unsaturated species. In agreement with this, we observed no significant changes in the mechanical strength or stiffness of the pure DPPC bilayer, which, based on its phase diagram, exists in the gel phase. We therefore attribute the absence of measurable IL effects to limited IL absorption by the highly ordered DPPC bilayer, as previously suggested. In contrast, the pure POPC and DOPC bilayers investigated in this study were in the Ld phase. The larger area per lipid (APL) in these bilayers (Table S3) provides greater free volume, allowing IL molecules to enter the membrane structure more readily. IL partitioning into the liquid phase of POPC has also been confirmed by neutron reflectometry^31^ and classical molecular dynamics simulations.^19,20,31,32^ Given the comparable APLs of POPC and DOPC, we assume that IL partitioning into these membranes is similar. Therefore, the observed differences in their mechanical properties are attributed to variations in IL–lipid interactions rather than differences in partitioning. Upon incubation with [C_6_mim]­[Cl], both RF and YM increased for the DOPC and POPC bilayers, indicating membrane strengthening and stiffening, respectively. The observed increases in mechanical strength and elasticity suggest that [C_6_mim]­[Cl] insertion exerts a stabilizing and structuring effect on the unsaturated lipid tails characteristic of DOPC and POPC. This likely reduces the conformational space available to the lipid tails, decreases fluidity (manifested as an increase in YM), and increases molecular surface density (reflected by an increase in RF). It is interesting to observe that the IL chain extends approximately until the kink in the lipid tail due to the unsaturated C–C bond, which might explain the bilayer’s stiffening mechanical response. The greater RF increase observed for POPC than for DOPC may be attributed to differences in their APL: the IL-induced surface density enhancement is more pronounced in the more tightly packed POPC bilayer, whereas in DOPC, where lipids are more widely spaced, the effect is smaller—consistent with the behavior previously reported for cholesterol in fluid DOPC membranes.^61^ The [C_6_mim]­[Cl]-induced increases in RF and YM observed for POPC and DOPC are consistent with trends reported for POPC in the presence of [C_4_mim]­[Cl],^26^ but differ from the effects of [C_*n*_mim]­[Cl] (*n* = 4, 8, 10) on DOPC bilayers.^23,27^

The system becomes more complex upon the addition of cholesterol. Cholesterol is generally understood to mitigate the disruptive effects of ILs;^62,63^ however, our observations reveal distinct trends. Initial incorporation of cholesterol into DPPC membranes induces partial fluidization, consistent with their phase diagram.^53^ We propose that this fluidizing effect enables greater IL penetration into the DPPC–cholesterol bilayer compared with the pure DPPC system, leading to further membrane disordering and a decrease in both RF and YM. The addition of 20 mol % cholesterol to POPC is expected to induce coexistence of Lo and Ld phases at 25 °C.^54^ In line with this, our data show an increase in the average RF relative to pure POPC, while YM remains essentially unchanged. This further confirms that RF and YM are independent parameters. Upon IL addition, both RF and YM decreased by −13 ± 7% and −19 ± 9%, respectively, indicating membrane weakening and softening. In contrast, DOPC with 20 mol % cholesterol remains largely in the Ld phase,^55^ resulting in the absence of a significant change in YM and even a decrease in RF upon cholesterol addition. In this system, IL treatment still produced a softening effect but was accompanied by an increase in RF, suggesting a complex interplay. Similar phenomena have been reported at high cholesterol concentrations, where membrane expansion triggers lipid headgroup tilting to maintain the ″umbrella effect″ that shields cholesterol’s hydrophobic core.^64^ The simultaneous increase in RF and decrease in YM observed here may arise from analogous headgroup tilting: additional IL insertion increases the area per lipid, promoting faster lipid tail dynamics, while lipid headgroups expand and tilt to preserve cholesterol shielding, thereby increasing molecular surface density. The observed mechanical weakening of POPC–cholesterol membranes, compared to pure POPC, may thus reflect limited free volume for IL insertion, leading to tail disordering. Conversely, in DOPC–cholesterol membranes—still largely liquid-disordered—the same IL insertion likely increases intermolecular spacing, exposing cholesterol cores to the aqueous phase and inducing compensatory headgroup tilting to maintain the ″umbrella″ effect.

Control experiments with NaCl solutions of equivalent ionic strength to [C_6_mim]­[Cl] confirmed that the observed effects cannot be attributed solely to ionic strength. Therefore, the organic component of the IL and its specific interactions with lipid bilayers is key in driving the IL–lipid interaction.

## Conclusion

AFM was employed to investigate the interactions between an imidazolium-based IL and lipid bilayers with varying degrees of acyl chain saturation. The model membranes were composed of four representative lipids commonly used in similar studies—three phosphatidylcholines (DPPC, DOPC, and POPC) and cholesterol. The IL, [C_6_mim]­[Cl], was selected based on previous evidence of its penetration beneath the interfacial zone of lipid bilayers^37^ and for the length of its chain respect to the lipids’ tails. Incubation at 10% of the IL’s CMC confirmed that this concentration was safe for membrane integrity, and a 30 min exposure was generally sufficient for the interaction to reach equilibrium.

Our results indicate that the IL effect depends strongly on the lipid saturation and cholesterol content, which impact lipid packing and conformational freedom and, in turn, might also control the IL partitioning between the lipid and aqueous phases. The response ranges from negligible effects in tightly packed, gel-phase membranes to pronounced stabilizing effects in loosely packed, liquid-disordered bilayers, where IL insertion ″pushes″ lipid molecules closer together, increasing mechanical stability. Cholesterol introduces additional complexity by imposing the requirement to maintain shielding of its rigid hydrophobic core, and represent an additional step toward better cell membrane models. For DPPC-Chol, incubation in [C_6_mim]­[Cl] leads to a decrease in RF and YM of −26 ± 9% and −30 ± 10%, respectively, while no effect is observed without cholesterol. For POPC, it leads to a + 30 ± 9% increase in RF in the absence of cholesterol, and to a −13 ± 7% decrease in RF and −19 ± 9% decrease in YM in the presence of cholesterol. For DOPC, a + 30 ± 14% increase in YM is observed without cholesterol, which reverts with the addition of cholesterol (−27 ± 5%) that, surprisingly, leads to a + 34 ± 9% increase in RF. Overall, our findings support previous reports suggesting that the effects of ILs on lipid membranes are highly system-dependent and governed by the physicochemical properties of both the IL and the lipid components.^30,65^ Together with the NaCl controls, our results highlight the importance of the IL’s organic part (e.g., IL alkyl chain length), which may allow tuning of its impact on the mechanical properties of supported lipid bilayers opening the way to potential applications.

Future studies should investigate the effect of the IL chain length on the mechanical response of lipid bilayers, and also further increase the complexity of the cell membrane models, to include sphingomyelin (to mimic raft domains), charged lipids (to mimic bacterial and eukaryotic cell membranes) and membrane proteins. Our results can form a solid base to better understand the mechanical response of more sophisticated cell membrane models to IL leading, in turn, to a better understanding of the IL–cell membrane interaction.

Furthermore, future studies should also quantify IL partitioning in the lipid phase for given IL concentrations in the aqueous phase and under well-defined conditions, and look for any correlation with the mechanistic effect of the IL on the bilayer. Techniques such as neutron reflectometry,^31^ X-ray reflectivity,^66^ and small-angle neutron scattering^67^ have already been successfully applied to study lipid bilayers and vesicles and could be used to calibrate more accessible approaches, such as zeta potential measurements,^68^ enabling extensive partitioning studies.

In summary, our results provide insights into how lipid saturation and cholesterol content drive the mechanical response of lipid bilayers to ILs, which can aid in understanding and predicting their effects on more complex biomembrane compositions. A deeper understanding of IL effects on biological membranes opens new opportunities in nanobiotechnology and nanomedicine, including applications in drug delivery and antimicrobial strategies.

## Supplementary Material



## Notes

Additional AFM topography maps; force spectroscopy results; supplementary tables summarizing mechanical properties of lipid bilayers in the presence and absence of [C_6_mim]­[Cl] and NaCl (PDF)
