# Supplementary material for: Lipid Saturation and Cholesterol Drive the Mechanical Response of Lipid Bilayer to Ionic Liquid: An Atomic Force Microscopy Study
Source: Langmuir. 2026 Mar 14;42(12):8405–15. doi: 10.1021/acs.langmuir.5c06231 (PMC13045010; doi:10.1021/acs.langmuir.5c06231)
Supplement: Supplementary file 1 [file la5c06231_si_001.pdf]

Supporting Information

Lipid Saturation and Cholesterol Drive the Mechanical Response of Lipid Bilayer to Ionic Liquid: An Atomic Force Microscopy Study

Alyona Yedelkina[1,2], Brian J. Rodriguez[1,2], Alessandro Podestà[3], Antonio Benedetto*[1,2,4]

[1]School of Physics, University College Dublin, Dublin D04 N2E5, Ireland
[2]Conway Institute of Biomolecular and Biomedical Research, University College Dublin, Dublin D04 N2E5, Ireland
[3]Department of Physics “Aldo Pontremoli”, University of Milano, 20133 Milan, Italy
[4]Department of Science, University of Roma Tre, 00146 Rome, Italy

*Corresponding author: Antonio Benedetto**
antonio.benedetto@ucd.ie

This Supporting Information file contains additional AFM topography maps, force spectroscopy results, and supplementary tables summarising mechanical properties of lipid bilayers in the presence and absence of [C_6mim][Cl] and NaCl.

Additional Figures

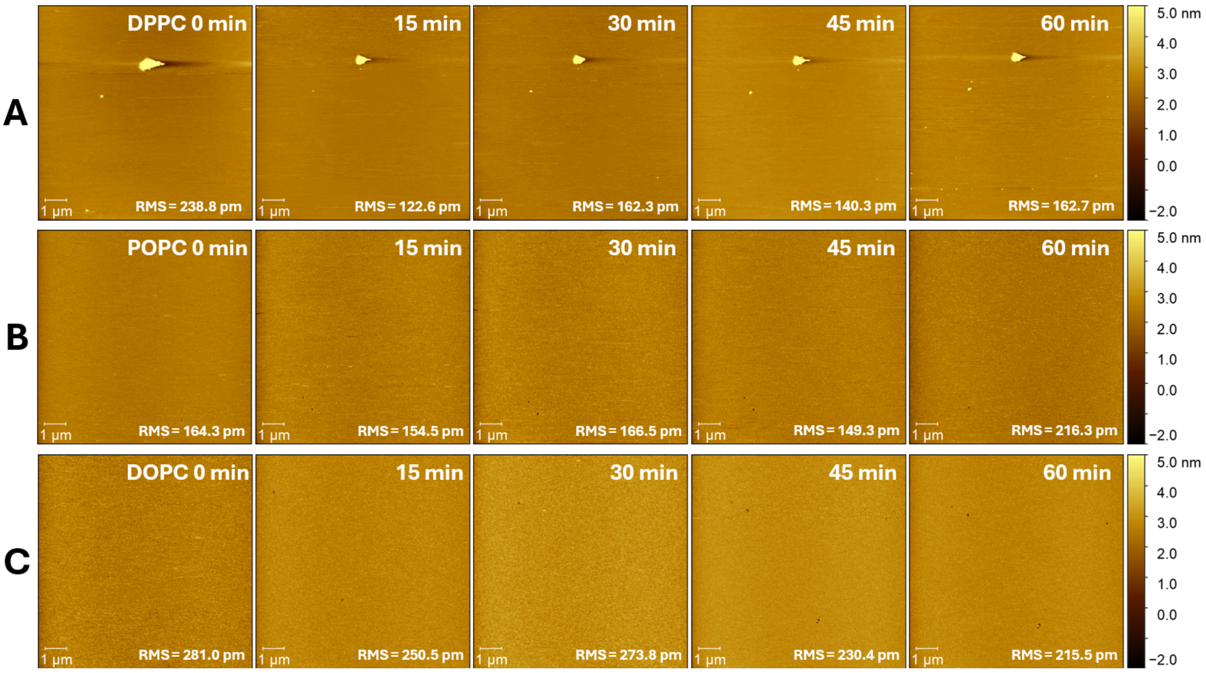

Figure S1: The representative time evolution of topographical maps of DPPC (A), POPC (B), and DOPC (C) bilayers upon incubation in [C_6mim][Cl].

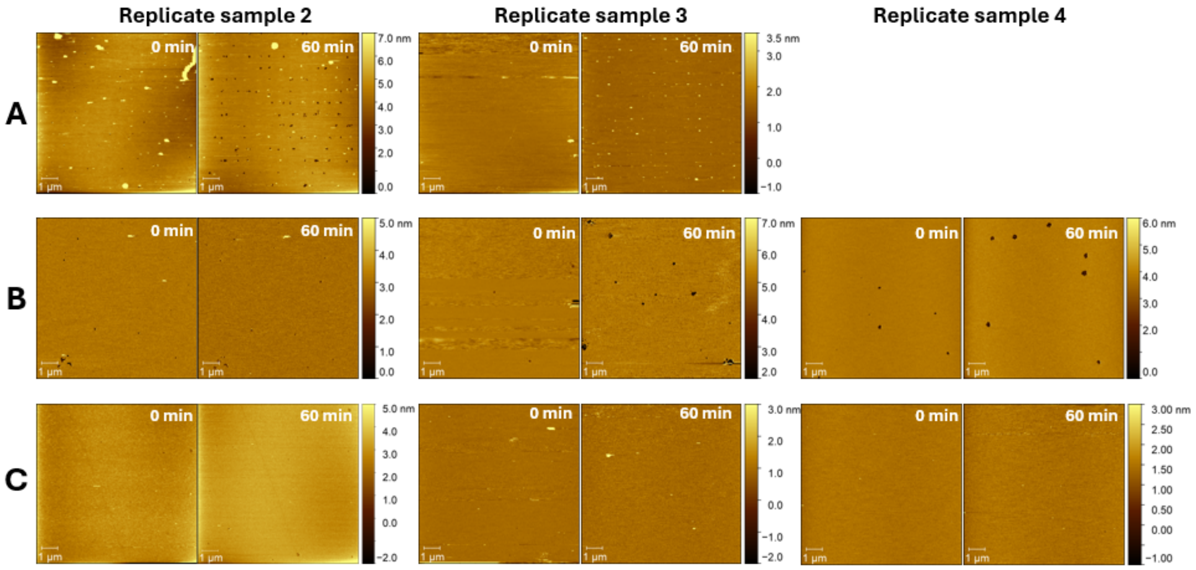

Figure S2: Topographical images of DPPC (A), POPC (B), and DOPC (C) bilayers before and 60 min after [C_6mim][Cl] addition for all remaining replicates. The respective replicate 1 samples are shown in Figure S1.

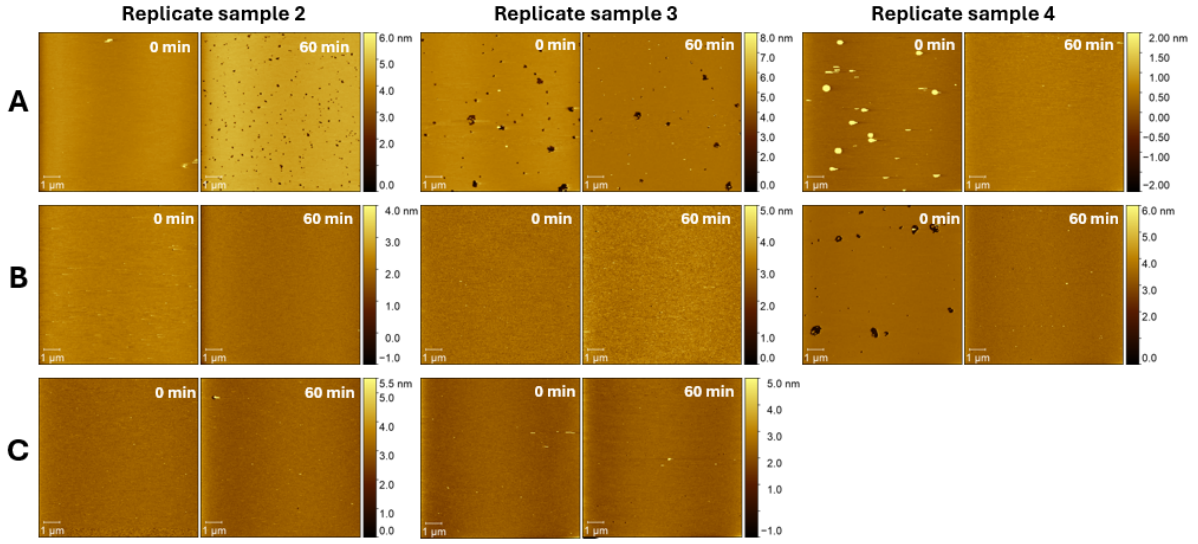

Figure S3: Topographical images of cholesterol-containing DPPC (A), POPC (B), and DOPC (C) bilayers before and 60 min after [C_6mim][Cl] addition for all remaining replicates. The respective replicate 1 samples are shown in the main text.

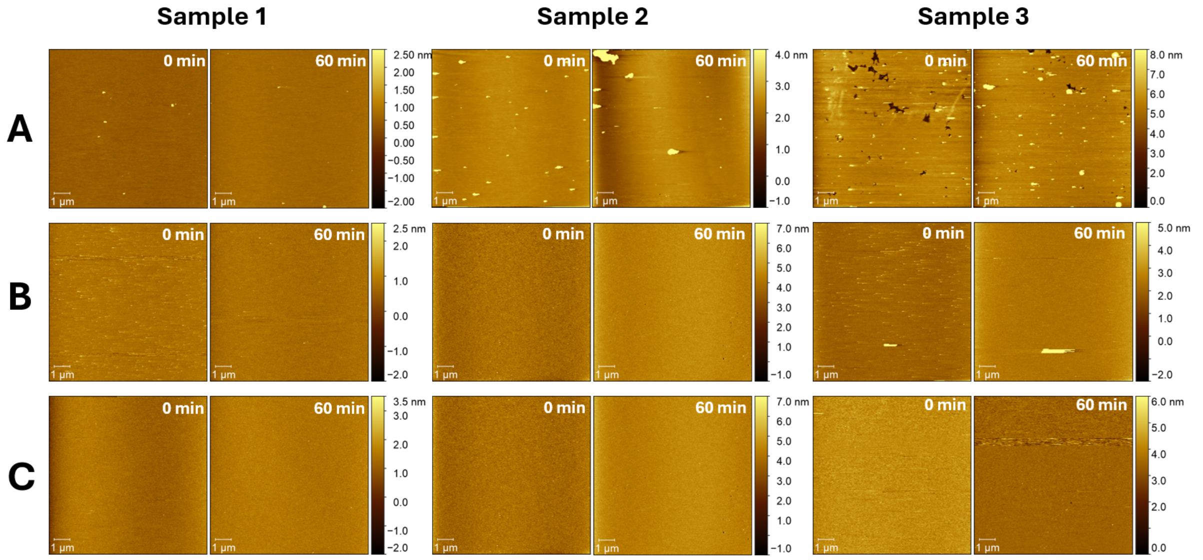

Figure S4: Topographical images of DPPC (A), POPC (B), and DOPC (C) bilayers before and 60 min after NaCl addition.

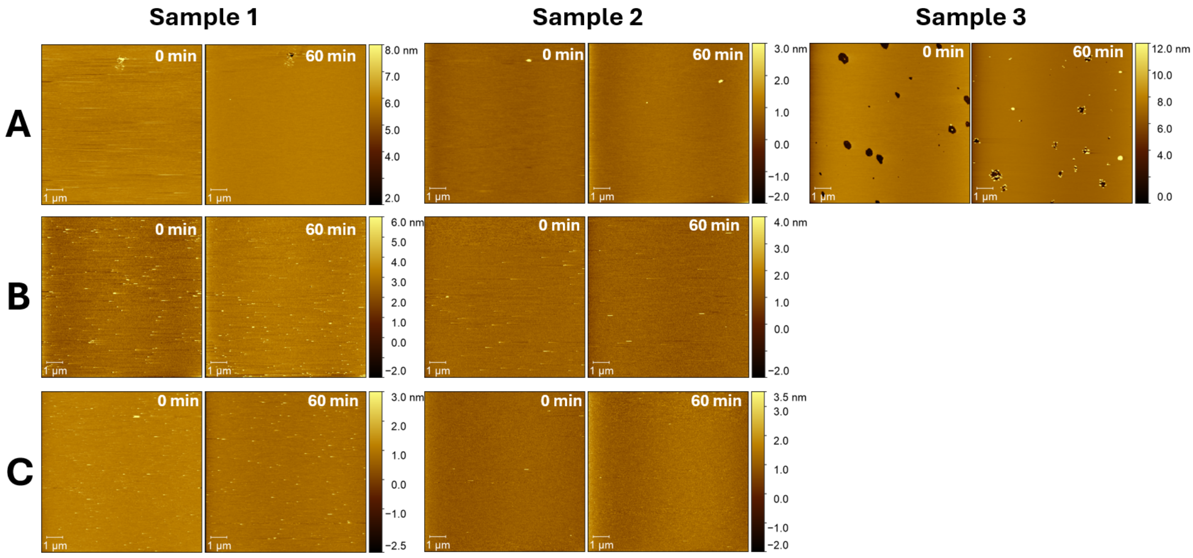

Figure S5: Topographical images of cholesterol-containing DPPC (A), POPC (B), and DOPC (C) bilayers before and 60 min after NaCl addition. In the presence of NaCl, cholesterol-containing POPC and DOPC membranes exhibited increased surface adhesion ("stickiness"), which complicated the AFM imaging.

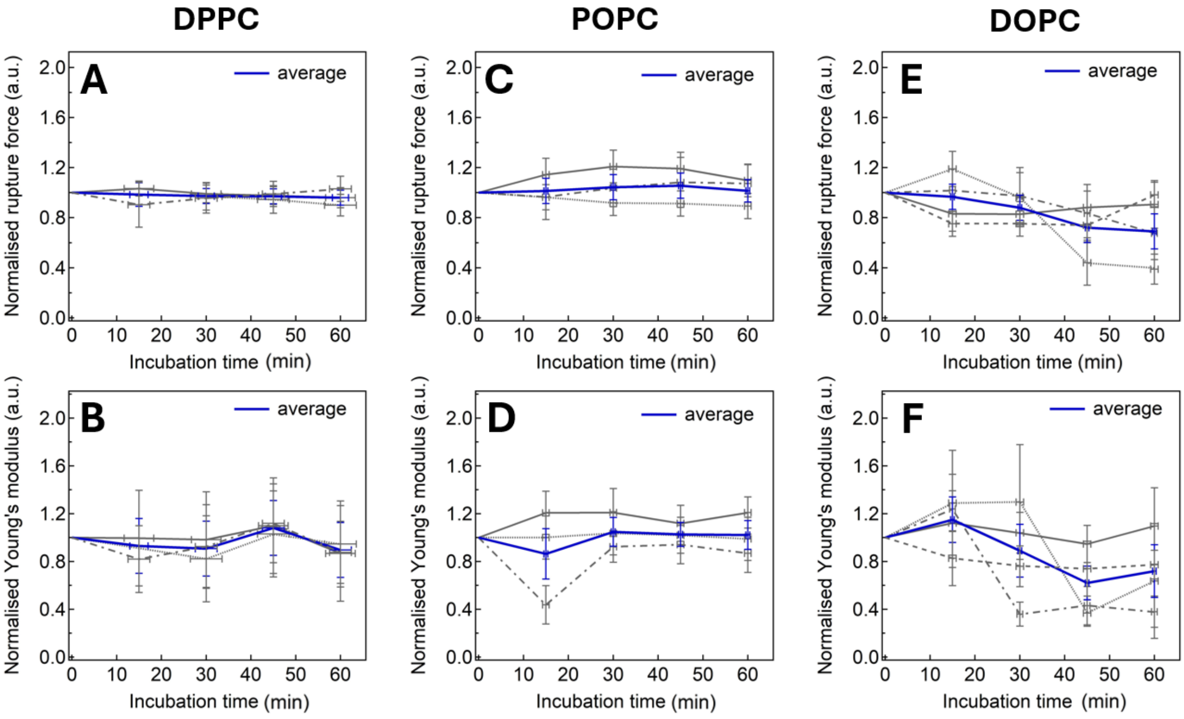

Figure S6: Time-dependence of NaCl-induced changes in 100% PC bilayers. Normalised RF (upper row) and YM (lower row) are shown. Panels A and B: DPPC; C and D: POPC; E and F: DOPC. Different sample replicates are plotted in grey. The average response across replicates is plotted in blue and is the same as that shown in Figure 7 in the main text.

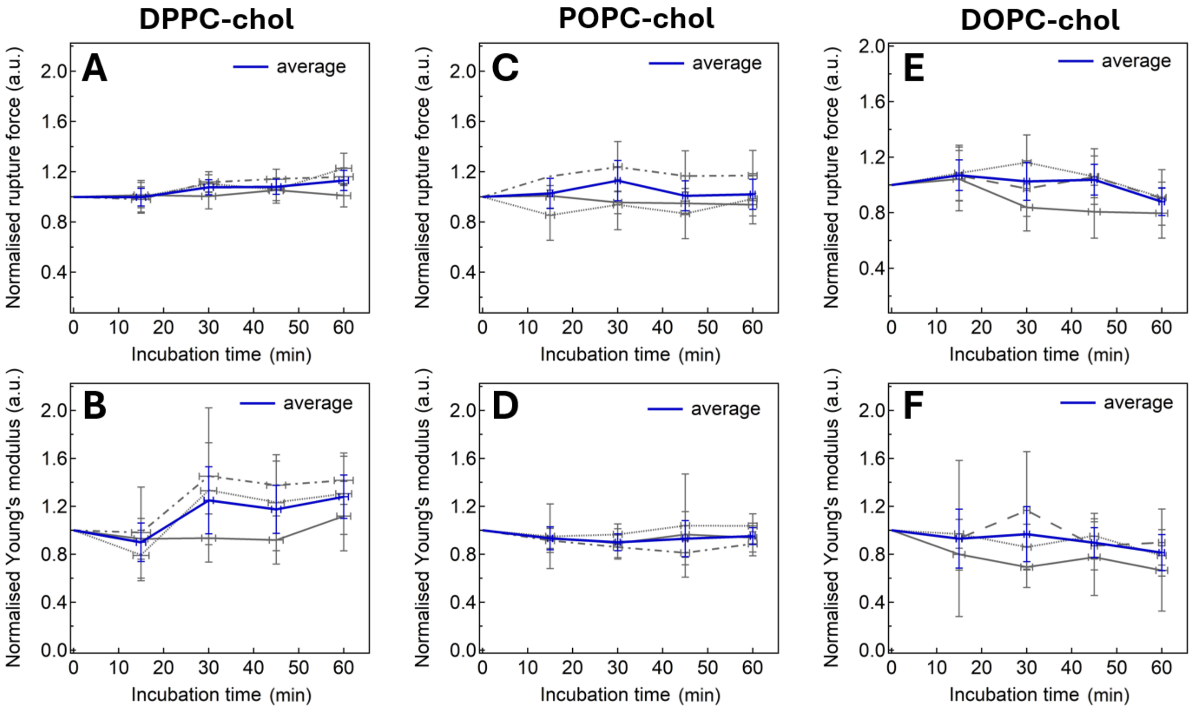

Figure S7: Time-dependence of NaCl-induced changes in PC–cholesterol bilayers. Normalised RF (upper row) and YM (lower row) are shown. Panels A and B: DPPC; C and D: POPC; E and F: DOPC. Different sample replicates are plotted in grey. The average response across replicates is plotted in blue and is the same as that shown in Figure 7 in the main text.

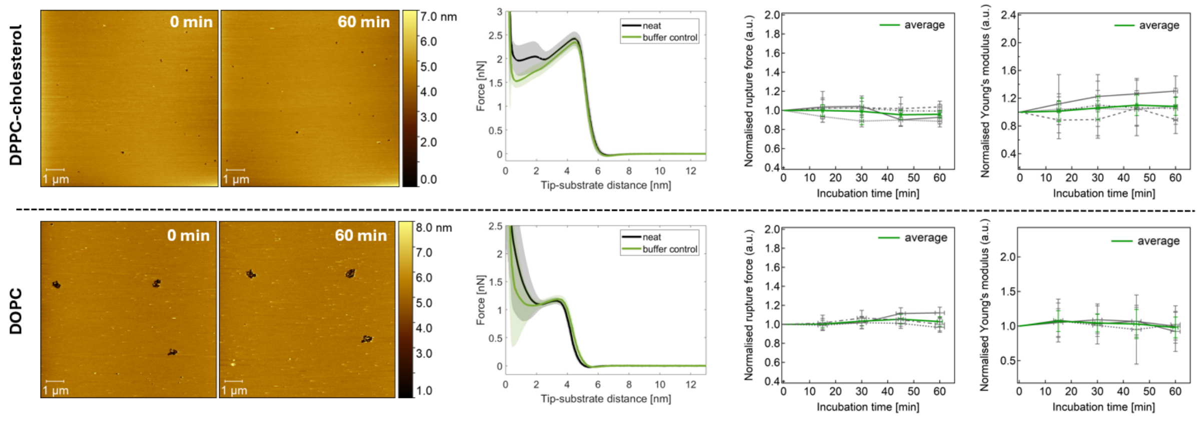

Figure S8: Representative topographical images, average force-distance curves, and time-dependency of RF and YM variation obtained for DPPC-cholesterol and DOPC bilayers in the 10 mM $CaCl_2$ buffer control experiments.

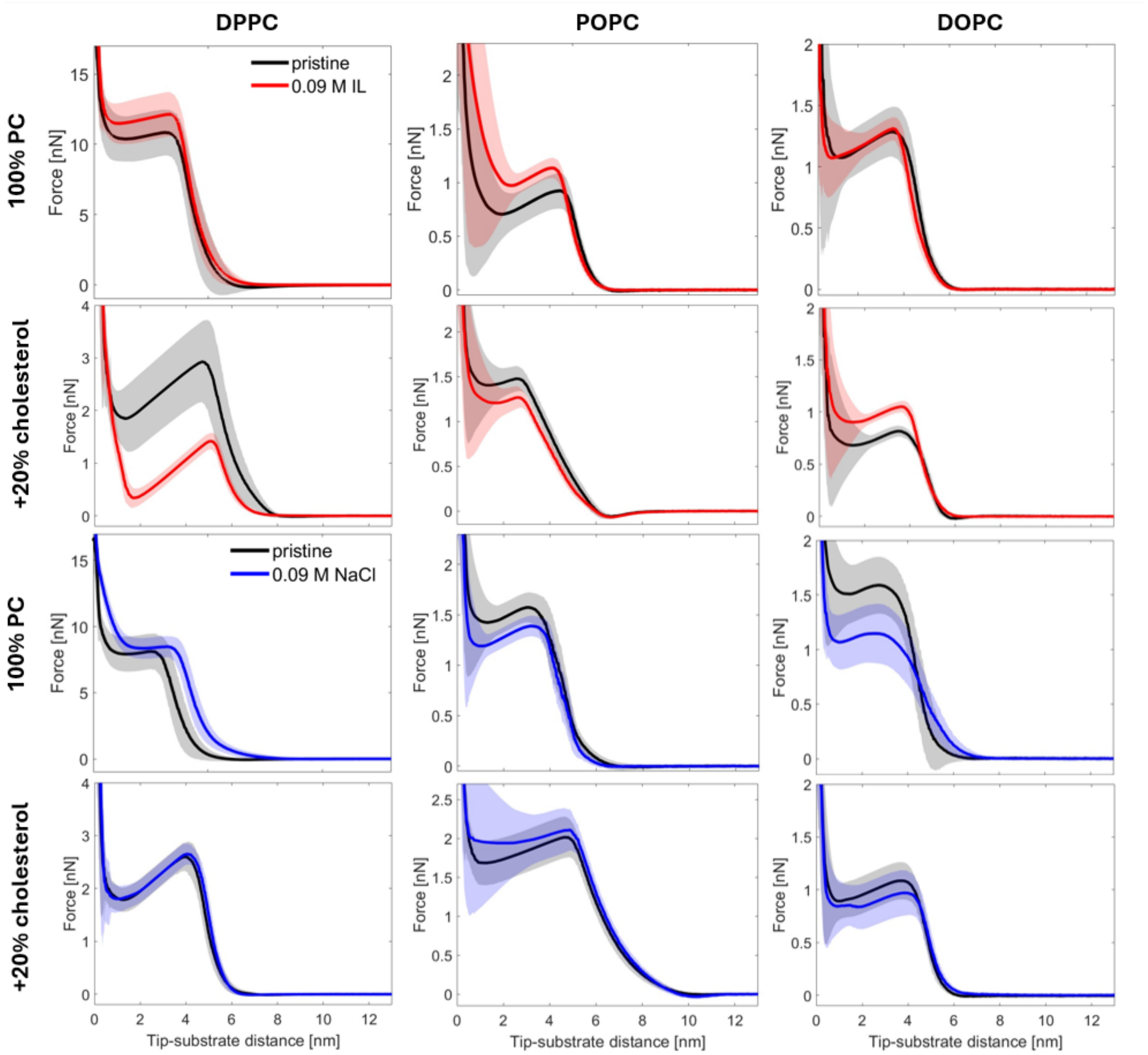

Figure S9: Representative average force-distance curves for all the bilayers compositions investigated. The bold lines indicate mean curves calculated from the 225 force-distance curves obtained from the 15 $\times$ 15 force maps, and the shaded regions show the associated standard deviations.

sample	indentation [nm]	thickness [nm]	R [nm]	chi	F_{ind} [nN]	delta	E [MPa]	std [MPa]	E normalised	std normalised	E_{corr} [MPa]	std_{corr} [MPa]	E_{corr} normalised	std_{corr} normalised
DPPC pristine 1	1.39	5.77	20	0.914	5	2.758	580.977	167.487	1.000	0.288	210.666	60.732	1.000	0.288
DPPC IL	2.16	6.25	20	1.052	6	3.248	486.677	115.549	0.838	0.199	149.832	35.574	0.711	0.169
DPPC-chol pristine 1	2.5	8	20	0.884	2	2.661	99.998	40.512	1.000	0.405	37.579	15.224	1.000	0.405
DPPC-chol IL	1.85	7.5	20	0.811	0.9	2.439	83.644	17.310	0.836	0.173	34.299	7.098	0.913	0.189
DPPC pristine 2	1.63	5.38	20	1.061	3.5	3.285	459.977	180.782	1.000	0.393	140.011	55.028	1.000	0.393
DPPC NaCl	2.73	7	20	1.056	5	3.263	402.848	122.058	0.876	0.265	123.444	37.402	0.882	0.267
DPPC-chol pristine 2	1.58	6.36	20	0.884	1.7	2.661	196.394	169.839	1.000	0.865	73.807	63.827	1.000	0.865
DPPc-chol NaCl	1.43	6.38	20	0.838	1.6	2.519	219.555	56.936	1.118	0.290	87.144	22.598	1.181	0.306

sample	indentation [nm]	thickness [nm]	R [nm]	chi	F_{ind} [nN]	delta	E [MPa]	std [MPa]	E normalised	std normalised	E_{corr} [MPa]	std_{corr} [MPa]	E_{corr} normalised	std_{corr} normalised
POPC pristine 1	1.23	6.43	20	0.771	0.5	2.325	60.167	14.018	1.000	0.233	25.873	6.028	1.000	0.233
POPC IL	1.44	6.43	20	0.835	0.6	2.509	62.147	11.017	1.033	0.183	24.774	4.392	0.958	0.170
POPC-chol pristine 1	1.8	5.9	20	1.017	0.8	3.118	33.614	5.565	1.000	0.166	10.781	1.785	1.000	0.166
POPC-chol IL	1.9	5.9	20	1.045	0.7	3.222	30.932	4.011	0.920	0.119	9.600	1.245	0.890	0.115
POPC pristine 2	1.65	6.5	20	0.884	0.5	2.661	27.635	4.604	1.000	0.167	10.387	1.730	1.000	0.167
POPC NaCl	1.6	6.5	20	0.870	0.4	2.618	27.334	5.194	0.989	0.188	10.441	1.984	1.005	0.191
POPC-chol pristine 2	3.23	9.2	20	0.874	1.2	2.629	31.821	4.265	1.000	0.134	12.106	1.623	1.000	0.134
POPC-chol NaCl	3.3	9.2	20	0.883	1.4	2.658	33.004	3.181	1.037	0.100	12.415	1.197	1.026	0.099

sample	indentation [nm]	thickness [nm]	R [nm]	chi	F_{ind} [nN]	delta	E [MPa]	std [MPa]	E normalised	std normalised	E_{corr} [MPa]	std_{corr} [MPa]	E_{corr} normalised	std_{corr} normalised
DOPC pristine 1	1.13	5.9	20	0.806	0.55	2.423	53.999	14.139	1.000	0.262	22.283	5.834	1.000	0.262
DOPC IL	1.2	5.7	20	0.859	0.6	2.584	46.217	7.266	0.856	0.135	17.883	2.811	0.803	0.126
DOPC-chol pristine 1	0.97	6.11	20	0.721	0.25	2.189	85.150	10.110	1.000	0.119	38.899	4.619	1.000	0.119
DOPC-chol IL	1.33	6.09	20	0.847	0.5	2.546	59.398	7.437	0.698	0.087	23.333	2.922	0.600	0.075
DOPC pristine 2	1.43	6.13	20	0.872	0.45	2.625	92.400	40.530	1.000	0.439	35.204	15.442	1.000	0.439
DOPC NaCl	1.75	6.7	20	0.883	0.45	2.658	34.853	12.630	0.377	0.137	13.112	4.751	0.372	0.135
DOPC-chol pristine 2	1.14	6.02	20	0.793	0.5	2.387	65.397	35.203	1.000	0.538	27.397	14.748	1.000	0.538
DOPC-chol NaCl	1.36	6.3	20	0.828	0.5	2.488	51.573	5.506	0.789	0.084	20.726	2.213	0.757	0.081

Figure S10: Bottom-effect correction factors calculated for the average force-distance curves shown in Figure S9. The table also compares absolute and normalised values of the uncorrected and corrected YM. The presence of a rigid underlying substrate, such as mica, can influence the apparent YM of thin soft films (e.g., lipid bilayers) measured by AFM indentation.[1] In this study, the bottom-effect correction was not applied; instead, force curves were fitted up to 20–30% of the bilayer thickness. Furthermore, all data were analysed in normalised form relative to the pristine bilayer values rather than as absolute moduli. As shown in the table, the normalised E and E_{corr} values are comparable, confirming the reliability of the reported data. R stays for the tip apex radius, F_{ind} for the force at the "indentation" (δ) reported in the table, *delta* for $\Delta = \sqrt{R\delta}/h$ (i.e., the Dimitriadis' bottom-effect parameter), h for the bilayer's height, E and E_{corr} for the apparent and bottom-effect-corrected YM, respectively, and std and std_{corr} for the corresponding standard deviations.

Supplementary Tables

Membrane composition	Average normalised rupture force (a.u.)	Average normalised Young's modulus (a.u.)
100% DPPC	1.06 ± 0.05	0.93 ± 0.08
80% DPPC - 20% cholesterol	0.74 ± 0.09	0.7 ± 0.10
100% POPC	1.30 ± 0.09	1.06 ± 0.13
80% POPC - 20% cholesterol	0.87 ± 0.07	0.81 ± 0.09
100% DOPC	1.07 ± 0.05	1.31 ± 0.14
80% DOPC - 20% cholesterol	1.34 ± 0.09	0.73 ± 0.05

Table S1: Normalised RF and YM averaged across all replicates between 30 and 60 minutes of incubation in the 0.09 M [C_6mim][Cl] solution. RF and YM were normalised relative to the pristine bilayer values.

Membrane composition	Average normalised rupture force (a.u.)	Average normalised Young's modulus (a.u.)
100% DPPC	0.97 ± 0.03	0.96 ± 0.13
80% DPPC - 20% cholesterol	1.1 ± 0.04	1.24 ± 0.13
100% POPC	1.04 ± 0.06	1.03 ± 0.07
80% POPC - 20% cholesterol	1.05 ± 0.08	0.93 ± 0.06
100% DOPC	0.76 ± 0.07	0.74 ± 0.11
80% DOPC - 20% cholesterol	0.98 ± 0.07	0.89 ± 0.10

Table S2: Normalised RF and YM averaged across all replicates between 30 and 60 minutes of incubation in the 0.09 M NaCl solution. RF and YM were normalised relative to the pristine bilayer values.

Lipid composition	Area per lipid (Å^2) at temperature T	T (K)
100% DPPC	46.8 ± 0.1 [2]	295
80% DPPC - 20% cholesterol	$\sim 48-49$ [3]	300
100% POPC	60.4 ± 0.9 [4]	300
80% POPC - 20% cholesterol	53.0 ± 0.7 [4]	300
100% DOPC	67.1 ± 0.5 [5]	323
80% DOPC - 20% cholesterol	58.7 ± 1.0 [5]	323

Table S3: Typical area per lipid values reported at temperature (T) for phosphatidylcholines in the studied membrane compositions.

References

[1] Emilios K. Dimitriadis, Ferenc Horkay, Julia Maresca, Bechara Kachar, and Richard S. Chadwick. Determination of elastic moduli of thin layers of soft material using the atomic force microscope. *Biophysical Journal*, 82(5):2798–2810, 2002.

[2] Dominik Drabik, Grzegorz Chodaczek, Sebastian Kraszewski, and Marek Langner. Mechanical properties determination of DMPC, DPPC, DSPC, and HSPC solid-ordered bilayers. *Langmuir*, 36(14):3826–3835, April 2020.

[3] Yin Wang, Paraskevi Gkeka, Julian E Fuchs, Klaus R Liedl, and Zoe Cournia. DPPC-cholesterol phase diagram using coarse-grained molecular dynamics simulations. *Biochim. Biophys. Acta*, 1858(11):2846–2857, November 2016.

[4] Alexandra Schumann-Gillett and Megan L O'Mara. The effects of oxidised phospholipids and cholesterol on the biophysical properties of POPC bilayers. *Biochim. Biophys. Acta Biomembr.*, 1861(1):210–219, January 2019.

[5] Mohammad Alwarawrah, Jian Dai, and Juyang Huang. A molecular view of the cholesterol condensing effect in DOPC lipid bilayers. *J. Phys. Chem. B*, 114(22):7516–7523, June 2010.
